# Endogenous endophthalmitis: a 9-year retrospective study at a tertiary referral hospital in Malaysia

**DOI:** 10.1186/s12348-018-0158-3

**Published:** 2018-10-11

**Authors:** Rosiah Muda, Valarmathy Vayavari, Deivanai Subbiah, Hamisah Ishak, Azian Adnan, Shelina Oli Mohamed

**Affiliations:** 1 Department of Opthalmology, Hospital Sultanah Nur Zahirah, Kuala Terengganu, Terengganu Malaysia; 2 Department of Opthalmology, Hospital Sungai Buloh, Sungai Buloh, Malaysia; 3 Department of Opthalmology, Hospital Selayang, Batu Caves, Malaysia; 4 Department of Opthalmology, Hospital Kuala Lumpur, Kuala Lumpur, Malaysia; 5 Department of Opthalmology, Hospital Shah Alam, Shah Alam, Malaysia

**Keywords:** Endogenous, Endophthalmitis, Diabetes mellitus, Bacteria, Fungal, Intravitreal injections, Vitrectomy, Visual acuity

## Abstract

**Background:**

The objective of this study was to determine the clinical presentation, systemic risk factors, source of infective microorganism, treatment outcomes, and prognostic indicators of endogenous endophthalmitis at a main tertiary referral hospital for uveitis in Malaysia. A retrospective review of medical records of 120 patients (143 eyes) with endogenous endophthalmitis over a period of 9 years between January 2007 and December 2015 was undertaken.

**Results:**

Identifiable systemic risk factors were present in 79.2%, with the majority related to diabetes mellitus (60.0%). The most common source of bacteremia was urinary tract infection (17.5%). A positive culture from ocular fluid or other body fluids was obtained in 82 patients (68.9%), and the blood was the highest source among all culture-positive results (42.0%). Gram-negative organisms accounted 42 cases (50.6%) of which *Klebsiella pneumonia* was the most common organism isolated (32.5%). Sixty-nine eyes (48.6%) were managed medically, and 73 eyes (51.4%) underwent vitrectomy. Final visual acuity of counting fingers (CF) or better was achieved in 100 eyes (73.0%). Presenting visual acuity of CF or better was significantly associated with a better final acuity of CF or better (*p* = 0.001).

**Conclusions:**

The visual prognosis of endogenous endophthalmitis is often poor, leading to blindness. As expected, gram-negative organisms specifically *Klebsiella pneumonia* were the most common organisms isolated. Urinary tract infection was the main source of infection. Poor presenting visual acuity was significantly associated with grave visual outcomes. A high index of suspicion, early diagnosis, and treatment are crucial to salvage useful vision.

## Background

Endogenous endophthalmitis (EE) occurs when infectious agents are hematogenously disseminated into the eye from a remote focus of infection. Even though this entity is relatively rare and accounts for approximately 2–15% of all cases of endophthalmitis [1–3], it is an ocular emergency and is potentially life-threatening. The causative organisms may vary depending on the geographical location. In Europe and the USA, *Streptococcus species*, *Staphylococcus aureus*, and other gram-positive bacteria account for two thirds of bacterial endogenous endophthalmitis cases and gram-negative isolates are found in only 32% of cases [3, 4]. In contrast, most cases of EE in East Asia are caused by gram-negative organisms especially *Klebsiella species* accounting for 80 to 90% of positive cultures [4, 5].

The outcome of endogenous endophthalmitis is often dismal. Sharma et al. reported that 60% of the eyes had a final visual acuity of hand motions or worse and as many as 29% required removals [6]. Hence, prompt diagnosis and management are essential if useful vision is to be preserved.

To the best of our knowledge, there is no large case series on endogenous endophthalmitis being reported yet from Malaysia. The current study was performed to determine the clinical profile of EE at a tertiary hospital while focusing on the clinical presentation, predisposing risk factors, source of infective microorganism, treatment outcomes and prognostic indicators.

## Methods

A retrospective observational study was conducted in Selayang Hospital which was the main national tertiary referral center for uveitis in Malaysia. We reviewed the medical records of patients with endogenous endophthalmitis who were seen or referred to our hospital over a period of 9 years between January 2007 and December 2015. The diagnosis of endogenous endophthalmitis was defined as the presence of iritis and vitritis on ophthalmic examination and one or more of the following: (1) constitutional symptoms and systemic infection; (2) positive cultures of vitreous, blood, or other body fluids; (3) presence of loculation, vitreous debris, or membranous debris on ultrasound; (4) lack of ocular trauma or ocular surgery within 1 year from onset of infection or evidence of primary external ocular infection such as infectious keratitis or filtering bleb infection.

Demographic details such as age, gender and race, presenting complaints, preexisting medical illnesses, predisposing risk factors, source of infection, laterality, visual acuity, ophthalmologic examination, ultrasound findings, microbiologic profiles, treatment modalities, and final visual outcomes were collected from medical records.

The study was done according to Malaysian Good Clinical Practice (MGCP) 2nd edition January 2004 and registered in National Medical Research Register (NMRR).

Data was analyzed using the Statistical Package for Social Science (SPSS) version 22.0. Descriptive data was expressed as mean ± standard deviation (SD) for numerical data, and categorical variables were presented in frequencies and percentages. Logistic regression analysis was used to determine the factors associated with good visual outcomes. The association between presenting and final visual acuity was also analyzed using the Pearson correlation coefficient, and visual acuities were converted to logarithm of the minimal angle of resolution (logMAR) scale. For visual acuity less than counting finger (CF), the following scales were used: CF = 2.00 LogMAR units, hand motion = 2.30 LogMAR units, light perception = 2.60 LogMAR units, and no light perception = 2.90 LogMAR units. A *P* value of < 0.05 was considered to be significant.

## Results

### Demographic data

A total of 143 eyes of 120 patients were included in this study. The age of patients ranged from 7 to 81 years, and the mean age at presentation was 52.6 ± 15.1 years. The racial distribution reflected the multiracial population in our country with 72 Malays (60.0%), 33 Chinese (27.5%), 13 Indian (10.8%), and 2 others (1.7%). There was a slight female predominance (61, 50.8%) compared to males (59, 49.2%).

### Systemic features

#### Systemic risk factors

At least one underlying medical illness was identified in 95 patients (79.2%). Diabetes mellitus was the most common medical illness (72, 60.0%), followed by renal failure (20, 16.7%), and 15 patients (12.5%) had solid organ tumor or hematologic malignancy. Six patients (5.0%) had liver disease and 3 patients (2.5%) were pregnant. Five patients (4.2%) were on systemic corticosteroids for underlying autoimmune diseases, and 1 was on systemic immunosuppressants.

#### Source of infection

A primary source of infection was identified in 90 patients (75.0%). Urinary tract infection (21, 17.5%) was the most common source of bacteremia followed by pulmonary infection (19, 15.8%), skin or soft tissue infection (17, 14.2%), and hepatobiliary infection (12, 10.0%). However, the source of infection could not be identified in 30 patients (25.0%), despite extensive systemic work-up and investigations (Table 1).

**Table 1:** Identifiable source of infection

Source of infection	No. of patients	Percent
Infected catheter	11	9.2
Urinary tract infection	21	17.5
Hepatobiliary infection	12	10.0
Lung infection	19	15.8
Meningitis	2	1.7
Infective endocarditis	1	0.8
Gastrointestinal infection	1	0.8
Genital infection	2	1.7
Skin or soft tissue infection	17	14.2
Septic arthritis	1	0.8
Diabetic foot ulcer	3	2.5
Nil	30	25.0

#### Ocular features

##### Ocular symptoms

Majority of patients had unilateral disease, 97 (80.8%), and involvement of the left eye (78, 54.5%) was more common. Blurring of vision (106, 74.1%), eye redness (52, 36.4%), eye pain or discomfort (42, 29.4%), and floaters (16, 11.2%) were ocular symptoms at presentation. The blurring of vision (85, 68.5%) was the most common presenting complaint, followed by eye redness (20, 16.1%) and eye pain or discomfort (14, 11.3%).

**Fig. 1:**
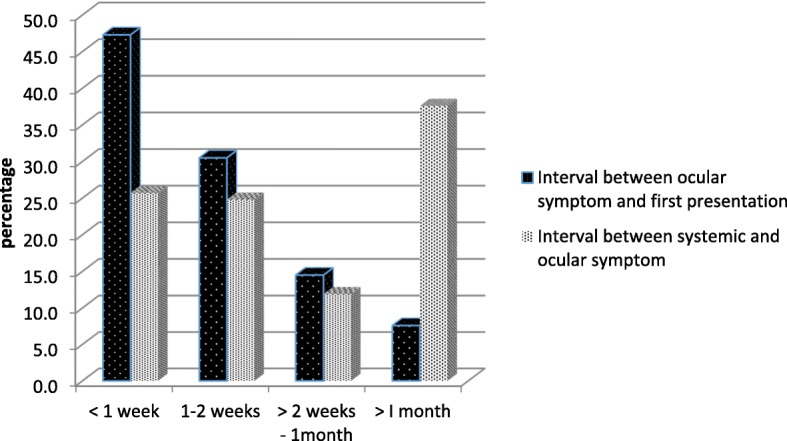
The interval between the onset of ocular symptom and first presentation and the duration between systemic and ocular symptom

The interval between the onset of ocular symptoms and the first presentation to an ophthalmologist was less than 1 week in 62 eyes (47.3%), 1 to 2 weeks in 40 eyes (30.5%), more than 2 weeks to 1 month in 10 eyes (14.5%), and more than 1 month in 10 eyes (7.6%). The interval was not available in 12 eyes which were from patients with no recorded ocular symptoms due to poor general medical condition or no documentation obtained from the medical records (Fig. 1).

Systemic symptoms were identified in 84 patients (70.0%). This data was not available in 36 patients (30.0%). The interval between the onset of systemic symptoms and the onset of ocular symptoms was identified in 101 eyes. The interval was less than 1 week in 26 eyes (25.7%), between 1 and 2 weeks in 25 eyes (24.8%), more than 2 weeks to 1 month in 12 eyes (11.9%), and more than 1 month in 38 eyes (37.6%) (Fig. 1).

##### Ocular findings

At presentation, based on the Standardization of Uveitis Nomenclature (SUN), the proportion of the eyes with anterior chamber cells better than grade 3 and grade 3 or worse was similar with 67 eyes (49.6%) and 68 eyes (50.4%) respectively. Twenty-seven eyes (19.0%) had hypopyon, and 42 eyes (29.6%) had fibrin. The fundal view was present in 64 out of 143 eyes (44.8%). Among this, 32 eyes (50.0%) had choroiditis or choroidal abscess, 11 eyes (17.2%) had retinitis, 6 (9.4%) had vasculitis, and 4 (6.3%) had optic disc swelling. Six eyes had a combination of choroiditis or choroidal abscess and retinitis and 2 eyes had choroiditis or choroidal abscess, vasculitis, and optic disc swelling.

Ultrasound findings were documented in 97 eyes. Fifty-five eyes (56.7%) had vitreous loculation, whereas subretinal or vitreous abscess and retinal detachment were found in 6 (6.2%) and 8 (8.2%) eyes respectively.

#### Microbiology

A positive culture from ocular fluid or other body fluids was obtained in 82 patients (68.9%). The culture result of 1 patient was not available as he had it done elsewhere.

**Fig. 2:**
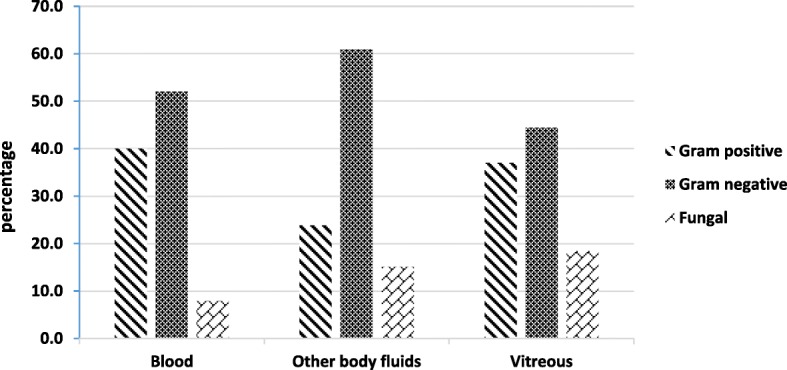
Blood, other body fluids, and vitreous culture organism

The blood was the highest source among all culture-positive results (50, 42.0%). Gram-negative organisms were more common (26 patients, 52.0%) than gram-positive organisms (20 patients, 40.0%). Four patients (8.0%) had a positive fungal culture from the blood (Fig. 2).

**Fig. 3:**
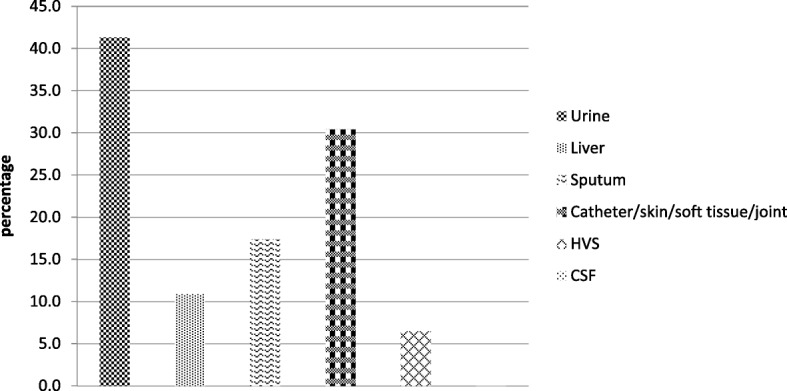
Other body fluid culture

Forty-six patients (38.7%) had at least one positive culture from other body fluids. Other body fluid cultures yielded gram-positive organisms in 11 patients (23.9%), gram-negative in 28 patients (23.3%), and fungal in 7 patients (15.2%). Nineteen patients (41.3%) had a positive urine culture; 14 (30.4%) had positive cultures from infected catheter, skin, soft tissue, or joint; 8 (17.4%) had positive sputum cultures; 5 (10.9%) had positive culture from liver abscess; 3 (6.5%) had positive high vaginal swab; and 1 patient (2.1%) had positive cerebrospinal fluid (Figs. 2 and 3).

Vitreous samples were obtained from 125 eyes. Vitreous culture was positive in 27 eyes (22.3%). Of these, 22 eyes (81.4%) had bacterial and 5 eyes (18.5%) had fungal isolates. Among bacterial isolates, 10 (37.0%) were gram-positive and 12 (44.4%) were gram-negative (Fig. 2).

The causative organisms cultured from the blood, vitreous, and other body fluids are summarized in Table 2.

**Table 2:** Microbial isolates from blood, other body fluids, and vitreous

Species	Blood (*n* = patient)	Other body fluids (*n* = patient)	Vitreous (*n* = eyes)
Culture positive	50 (42.0%)	46 (38.7%)	27 (22.3%)
Culture negative	69 (58.0%)	73 (61.3%)	94 (77.7%)
Gram-positive organism	20 (40.0%)	11 (23.9%)	10 (37.0%)
*Staphylococcus aureas*	13 (26.0%)	7 (15.2%)	5 (18.5%)
MRSA	3 (6.0%)	2 (4.3%)	2 (7.4%)
*Staphylococcus* coagulase -ve	2 (4.0%)	0 (0.0%)	2 (7.4%)
*Streptococcus* sp.	2 (4.0%)	2 (4.3%)	1 (3.7%)
Gram-negative organism	26 (52.0%)	28 (60.9%)	12 (44.4%)
*Klebsiella pneumonia*	17 (34.0%)	19 (41.3%)	8 (29.6%)
*Pseudomonas aeruginosa*	2 (4.0%)	4 (8.7%)	3 (11.1%)
*Escherichia coli*	1 (2.0%)	3 (6.5%)	0 (0.0%)
*Acinebacter* sp.	1 (2.0%)	2 (4.3%)	0 (0.0%)
*Enterobacter intermedius*	1 (2.0%)	0 (0.0%)	0 (0.0%)
*Bukholderia cepacia*	1 (2.0%)	0 (0.0%)	1 (3.7%)
*Bukholderia pseudomallei*	1 (2.0%)	0 (0.0%)	0 (0.0%)
*Elizabethkingia meningosepticum*	1 (2.0%)	0 (0.0%)	0 (0.0%)
*Mycoplasma pneumonia*	1 (2.0%)	0 (0.0%)	0 (0.0%)
Fungal	4 (8.0%)	7 (15.2%)	5 (18.5%)
*Candida albicans*	1 (2.0%)	5 (10.9%)	1 (3.7%)
*Candida tropicalis*	2 (4.0%)	1 (2.2%)	0 (0.0%)
*Penicillium* sp.	0 (0.0%)	0 (0.0%)	2 (7.4%)
*Phanerochaeta chrysosporium*	0 (0.0%)	0 (0.0%)	1 (3.7%)
*Xylariaceae* sp.	0 (0.0%)	0 (0.0%)	1 (3.7%)
Fungal (species not available)	1 (2.0%)	1 (2.2%)	0 (0.0%)

The microbiology of causative organisms is summarized in Table 3.

**Table 3:** Microbiology of causative organism in endogenous endophthalmitis

	Number	Percent
Gram-positive bacteria	27	32.5
*Staphylococcus aureas*	17	20.5
MRSA	3	3.6
*Staphylococcus* coagulase -ve	4	4.8
*Streptococcus* sp.	3	3.6
Gram-negative bacteria	42	50.6
*Klebsiella pneumonia*	27	32.5
*Pseudomonas aeruginosa*	5	6.0
*Escherichia coli*	3	3.6
*Acinebacter* sp.	2	2.4
*Enterobacter intermedius*	1	1.2
*Bukholderia cepacia*	1	1.2
*Bukholderia pseudomallei*	1	1.2
*Elizabethkingia meningosepticum*	1	1.2
*Mycoplasma pneumonia*	1	1.2
Fungal	14	16.9
*Candida albicans*	5	6.0
*Candida tropicalis*	3	3.6
*Penicillium* sp.	2	2.4
*Phanerochaeta chrysosporium*	1	1.2
*Xylariaceae* sp.	1	1.2
Fungal (species not available)	2	2.4

#### Treatment

All patients were treated with systemic antibiotics or antifungal agents aimed at the source of infection and presumed causative organism. One hundred nineteen cases (85.6%) were on systemic antibiotics, and more than half (55.1%) were treated with ciprofloxacin either as monotherapy or in combination with other antibiotics. There was no statistically significant correlation between systemic ciprofloxacin and final visual outcomes (*p* = 0.68). Systemic steroids were not used in any of the patients. In addition, 126 eyes (88.7%) received intravitreal antibiotics (vancomycin and ceftazidime or amikacin) or antifungal (amphotericin B) injections or both. The injections were repeated in 75 eyes (59.5%). Intravitreal injection was not given in 16 cases (11.3%), and 10 of them (62.5%) had relatively good presenting visual acuity of 6/24 or better and mild vitritis. They were treated with systemic antibiotics or antifungals with close monitoring. No intravitreal injection of steroids was given to any eye. Overall, 69 eyes (48.6%) were managed medically either with systemic or intravitreal antibiotics or antifungals or both.

Out of 143 eyes, 73 (51.4%) underwent vitrectomy. Early vitrectomy was performed within 2 weeks from diagnosis in 38 eyes (52.1%). Silicone oil was injected in 35 eyes (47.9%), gas in 3 eyes (4.1%), air in 19 eyes (26.0%), and no intraocular tamponade was used in 16 eyes (21.9%).

#### Outcomes

The most common presenting visual acuity was between 6/60 and counting finger (CF) (48 eyes, 34.0%), followed by hand motion (38, 27.0%) and perception of light (22 eyes, 15.6%). Only 16 eyes (11.3%) and 14 eyes (9.9%) had presented visual acuity between 6/6 to 6/18 and 6/24 to 6/36 respectively. Overall, 56% had vision of CF or better at presentation. Vision was not available in 2 patients (1.4%) who could not cooperate during a vision test. Final visual acuity was available in 138 eyes (96.5%). Three patients were transferred to their original hospitals for the continuation of treatment and follow-up. One patient defaulted follow-up, and another patient passed away due to sepsis and multiorgan failure.

After treatment, 100 eyes (73.0%) achieved final visual acuity of CF or better. Ten eyes developed panophthalmitis, and 6 eyes required evisceration. Table 4 summarizes the logistic regression analysis results of patient characteristics that may predict a good visual outcome (CF or better).

**Table 4:** Prognostic factors associated with good visual outcomes

Prognostic factor (referent)	Crude odds ratio (95% CI)	*p* value^a^	Adjusted odds ratio (95% CI)	*p* value^b^
Gender (male)				
Female	2 (0.92, 4.37)	0.079		
Age (0–50 years)				
> 50 years	0.85 (0.39, 1.86)	0.679		
Medical illness (yes)				
No	1.60 (0.55, 4.63)	0.383		
DM (yes)				
No	2.12 (0.93, 4.85)	0.071		
Presenting VA (≥ CF)				
< CF	0.10 (0.04, 0.25)	0.000	0.09 (0.021, 0.384)	0.001
Fundus view (yes)				
No	0.15 (0.06, 0.38)	0.000	0.337 (0.068, 1.675)	0.184
Source of infection (yes)				
No	2.61 (0.93, 7.37)	0.062		
Culture (positive)				
Negative	1.29 (0.54, 3.07)	0.566		
Organism (gram+)				
Gram−	0.965 (0.275–3.386)	0.956		
Fungal	0.185 (0.019, 1.848)	0.151		
Intravitreal antibiotic (yes)				
No	2.85 (0.62, 13.2)	0.234		
Vitrectomy (yes)				
No	1.13 (0.53–2.41)	0.751		
Early vitrectomy (≤ 2 weeks)				
> 2 weeks	1.69 (0.59–4.82)	0.327		

The clinical summary of patients were summarized in Table 5

^a^Univariate logistic regression

^b^Only factors associated with good visual outcome in multivariate logistic regression model

**Table 5:** Clinical summary of endogenous endophthalmitis in the current study

Case	Age/sex/eye	Systemic risk factors	Initial VA	Culture			Organism	Source of infection	Systemic antibiotics/antifungal	IVT abk/antifungal	Vitrectomy	Final VA
				Blood	Other body fluids	Vitreous						
1	49/M/R	–	CF	+	+ Sputum	−	*Acinetobacter* sp.	Skin infection	Ciprofloxacin	Yes	Yes	6/18
2	76/F/R	–	PL	−	+ Skin	−	*Candida albicans*	Skin infection	Fluconazole	Yes	Yes	NPL
3	49/M/L	DM	4/60	−	+ Skin	−	*Staphylococcus aureus*	Skin infection	Unasyn	Yes	No	6/36
4	46/F/L	–	NAV	−	+ Sputum	−	*Acinetobacter* sp.	Lung infection	Ceftriaxone, sulperazone	Yes	No	NAV
5	39/F/L	Leukemia	6/36	−	−	−	–	–	Amphotericin, fluconazole	Yes	yes	6/9
6	71/M/L	–	CF	+	−	−	*Klebsiella pneumonia*	UTI	Cefuroxime	Yes	No	6/24
7	56/F/R	DM	NPL	−	−	−	–	Lung infection	Ciprofloxacin	Yes	No	NPL
8	56/M/L	Leukemia	6/60	+	−	−	*Enterobacter intermedius*	–	Vancomycin, moxifloxacin	Yes	No	HM
9	26/M/R	–	6/36	+	−	Not done	*Staphylococcus aureus*	IE	Cloxacillin, gentamicin	No	No	6/36
10	26/M/L	–	6/9	+	−	Not done	*Staphylococcus aureus*	IE	Cloxacillin, gentamicin	No	No	6/6
11	61/M/R	DM	CF	+	+ Urine	−	*Candida albicans*	UTI	Fluconazole, voriconazole	Yes	Yes	6/9
12	61/M/L	DM	6/36	+	+ Urine	−	*Candida albicans*	UTI	Fluconazole, voriconazole	Yes	Yes	6/9
13	60/M/R	DM	PL	−	+ Liver, + sputum	−	*Klebsiella pneumonia*	HPB infection	Ciprofloxacin, cefuroxime, metronidazole	Yes	Yes	PL
14	60/M/L	DM	PL	−	+ Liver, + sputum	−	*Klebsiella pneumonia*	HPB infection	Ciprofloxacin, cefuroxime, metronidazole	Yes	No	NPL
15	49/M/R	DM	HM	−	+ Urine	−	*Staphylococcus aureus*	DFU	Ciprofloxacin	Yes	Yes	CF
16	49/M/L	DM	HM	−	+ Urine	−	*Staphylococcus aureus*	DFU	Ciprofloxacin	Yes	Yes	NPL
17	52/F/L	DM	CF	−	−	−	–	–	Ciprofloxacin, vancomycin	Yes	No	CF
18	57/M/L	DM, renal failure	CF	−	+ Infected catheter	+	*Pseudomonas aeruginosa*	DFU	Ciprofloxacin, ceftazidime, meropenem	Yes	Yes	NPL
19	38/F/L	DM	HM	−	−	−	–	HPB infection	Ciprofloxacin	Yes	Yes	HM
20	71/F/R	DM	CF	+	−	−	*Staphylococcus* coagulase -ve	Lung infection	Cefuroxime	Yes	No	6/60
21	49/F/L	Renal failure on steroid	CF	−	−	−	–	–	Fluconazole	Yes	Yes	6/12
22	68/M/L	DM, malignancy	6/12	−	+ Sputum	Not done	*Pseudomonas aeruginosa*	Lung infection	Ceftriaxone, cefuroxime	No	No	6/9
23	56/M/L	DM, renal failure, hydronephrosis	4/60	+	−	−	*Staphylococcus aureus*	Infected catheter	Cloxacillin	Yes	No	4/60
24	30/F/R	DM	PL	−	+ Urine	+	*Klebsiella pneumonia*	UTI	Vancomycin, ceftazidime	Yes	Yes	PL
25	65/F/R	–	6/60	−	−	−	–	UTI	NAV	Yes	Yes	6/24
26	19/F/R	SLE with lupus nephritis, on steroid	6/6	+	−	−	*Staphylococcus aureus*	Infected catheter	Vancomycin	Yes	No	6/18
27	75/F/R	DM	6/18	−	+ Sputum	Not done	*Klebsiella pneumonia*	Lung infection	Ciprofloxacin	No	No	6/24
28	75/F/L	DM	CF	−	+ Sputum	−	*Klebsiella pneumonia*	Lung infection	Ciprofloxacin	Yes	Yes	PL
29	51/F/R	DM	HM	−	+ HVS	−	*Candida albicans*	–	Fluconazole	Yes	Yes	6/9
30	53/F/L	–	HM	+	−	−	*Staphylococcus aureus*	–	Ciprofloxacin	Yes	Yes	CF
31	49/M/L	DM	HM	NAV	NAV	−	NAV	HPB infection	NAV	Yes	Yes	CF
32	19/M/R	–	HM	−	−	Not done	–	Meningitis	Ciprofloxacin	Yes	No	NAV
33	65/F/L	CNS lymphoma	CF	−	−	−	–	–	Ciprofloxacin	Yes	Yes	CF
34	77/M/R	–	CF	−	−	−	–	–	Ciprofloxacin, fluconazole	Yes	Yes	6/36
35	27/F/R	–	6/24	−	−	Not done	–	Lung infection	NAV	No	No	6/9
36	27/F/L	–	6/24	−	−	Not done	–	Lung infection	NAV	No	No	6/9
37	56/F/R	–	3/60	+	−	Not done	*Staphylococcus aureus*	UTI	C-penicillin, unasyn	No	No	6/9
38	62/M/R	DM	6/12	+	+ Urine	Not done	*Streptococcus* sp.	UTI	Piperacillin-tazobactam	Yes	No	6/9
39	62/M/L	DM	PL	+	+ Urine	−	*Streptococcus* sp.	UTI	Piperacillin-tazobactam	Yes	No	6/18
40	34/M/R	Renal failure	HM	−	+ Urine	NAV	*Klebsiella pneumonia*	UTI	Sulperazone	Yes	No	3/60
41	63/M/L	DM	CF	−	+ Sputum	Not done	*Klebsiella pneumonia*	Lung infection	Trimethoprime, sulphamethoxazole	No	No	CF
42	30/F/R	–	6/60	−	−	−	–	Skin infection	Cloxacillin	Yes	No	6/6
43	30/F/L	–	6/24	−	−	Not done	–	Skin infection	Cloxacillin	Yes	No	6/6
44	61/F/R	DM, sigmoid colon carcinoma	CF	−	−	−	–	Infected catheter	Ciprofloxacin, fluconazole	Yes	Yes	6/36
45	40/M/L	–	HM	−	−	−	–	HPB infection	Ciprofloxacin, meropenem	Yes	Yes	NPL
46	53/M/R	DM	HM	−	−	−	–	Lung infection	Cefoperazone	Yes	No	6/60
47	53/M/L	DM	HM	−	−	−	–	Lung infection	Ceftriaxone	Yes	No	HM
48	57/M/R	DM	HM	−	−	+	*Staphylococcus aureus*	UTI	Vancomycin	Yes	Yes	HM
49	57/M/L	DM	3/60	−	−	+	*Staphylococcus aureus*	UTI	Vancomycin	Yes	Yes	NPL
50	70/M/R	DM, hydronephrosis	HM	+	−	−	*Staphylococcus aureus*	Infected catheter	Cloxacillin, gentamicin	Yes	No	6/18
51	70/M/L	DM, hydronephrosis	HM	+	−	−	*Staphylococcus aureus*	Infected catheter	Cloxacillin, gentamicin	Yes	No	CF
52	19/M/L	Takayashu arteritis, renal failure on steroid	2/60	−	−	−	–	–	Ciprofoxacin, ceftazidime	Yes	No	6/18
53	56/F/R	DM	6/18	+	+ Liver	−	*Klebsiella pneumonia*	HPB infection	Cefuroxime	Yes	No	6/18
54	56/M/R	DM	HM	+	−	−	MRSA	Infected catheter	Ciprofloxacin	Yes	No	CF
55	56/M/L	DM	CF	+	−	−	*Klebsiella pneumonia*	Lung infection	Ceftazidime	Yes	No	NPL
56	57/M/R	–	CF	−	−	−	–	–	Ciprofloxacin	Yes	No	6/12
57	57/M/L	–	6/12	−	−	Not done	–	–	Ciprofloxacin	Yes	No	6/12
58	38/F/L	–	6/9	−	+ Urine	−	*Escherichia coli*	UTI	Ceftazidime	No	No	6/9
59	55/M/R	DM	CF	−	+ Infected catheter	−	*Staphylococcus aureus*	Skin infection	Ceftriaxone	Yes	No	CF
60	7/M/R	Leukemia	6/36	−	+ Infected catheter	−	Fungal*	Septic arthritis	Itraconazole	Yes	Yes	6/9
61	42/F/R	–	CF	+	−	−	*Bukholderia pseudomallei*	–	Ciprofloxacin	Yes	No	6/24
62	63/F/R	DM	NAV	+	+ Urine/infected catheter	Not done	*Klebsiella pneumonia*	Skin infection	Ciprofloxacin	No	No	NPL
63	71/F/L	–	HM	−	−	NAV	–	Lung infection	Ciprofloxacin	NAV	NAV	NAV
64	22/F/L	Leukemia	6/24	+	−	Not done	Fungal*	–	Voriconazole	No	No	6/18
65	45/M/L	DM, renal failure	NPL	+	+ Infected catheter	−	*Staphylococcus aureus*	Infected catheter	Cloxacillin	Yes	No	NPL
66	48/M/R	DM	CF	+	−	−	*Klebsiella pneumonia*	Lung infection	Ceftriaxone, amoxicillin-clavulanic acid	Yes	Yes	6/60
67	31/M/L	–	CF	−	−	−	–	–	Ciprofloxacin	Yes	Yes	6/6
68	54/M/L	–	6/60	+	+ Sputum, CSF	+	*Klebsiella pneumonia*	Lung infection	Ceftriaxone, imipenem	Yes	No	NPL
69	55/M/R	DM, renal failure	HM	+	+ Infected catheter	−	*Staphylococcus aureus*	Skin infection	Ciprofloxacin	Yes	Yes	6/60
70	55/M/L	DM, renal failure	HM	+	+ Infected catheter	−	*Staphylococcus aureus*	Skin infection	Ciprofloxacin	Yes	Yes	6/36
71	14/F/L	–	6/36	−	−	Not done	–	–	Ceftazidime, trimethoprime, sulphamethoxazole	No	No	6/18
72	42/F/L	–	6/60	−	−	−	–	HPB infection	Ciprofloxacin, Ceftazidime	Yes	Yes	CF
73	30/F/R	–	6/12	−	+ HVS	−	*Steroptococcus* sp.	Genital infection	c-penicillin	Yes	No	6/12
74	67/F/L	DM	HM	−	−	−	–	Lung infection	Ceftazidime	Yes	No	NAV
75	65/M/L	–	CF	−	+ Liver	−	*Klebsiella pneumonia*	HPB infection	Ciprofloxacin, imipenem	Yes	Yes	CF
76	34/F/R	Leukemia	PL	+	−	−	*Candida tropicalis*	–	Voriconazole	Yes	No	NPL
77	34/F/L	Leukemia	CF	+	−	−	*Candida tropicalis*	–	Voriconazole	Yes	Yes	6/60
78	58/M/R	DM, renal failure	NPL	+	+ Urine	+	*Pseudomonas aeruginosa*	UTI	Ciprofloxacin, metronidazole	No	No	NPL
79	55/F/R	DM	6/60	+	−	+	*Bukholderia cepacia*	Meningitis	Ceftazidime	Yes	No	6/36
80	25/M/L	–	PL	+	−	+	*Steroptococcus* sp.	–	Cloxacillin	Yes	Yes	NPL
81	48/F/L	–	6/60	−	−	−	–	–	Ceftazidime	Yes	No	6/18
82	52/F/L	DM	CF	+	+ Urine	−	*Pseudomonas aeruginosa*	UTI	Ciprofloxacin	Yes	Yes	CF
83	55/F/L	DM	HM	+	−	−	*Klebsiella pneumonia*	Lung infection	Amoxicillin-clavulanic acid, azithromycin	Yes	Yes	CF
84	74/M/L	DM, renal carcinoma	CF	−	−	−	–	Skin infection	Ciprofloxacin, fluconazole	Yes	Yes	6/24
85	37/F/R	DM	HM	+	−	−	–	Lung infection	Ciprofloxacin	Yes	Yes	5/60
86	66/F/R	DM	PL	−	+ Infected catheter	+	*Klebsiella pneumonia*	Skin infection	Ciprofloxacin	Yes	Yes	CF
87	66/F/L	DM	PL	−	+ Infected catheter	+	*Klebsiella pneumonia*	Skin infection	Ciprofloxacin	Yes	No	CF
88	50/F/R	DM	HM	+	−	−	*Staphylococcus* coagulase -ve	UTI	Ciprofloxacin	Yes	No	6/9
89	65/M/R	DM, alcoholic liver disease	CF	−	+ Urine	−	*Candida albicans*	HPB infection	Fluconazole	Yes	Yes	CF
90	65/M/L	DM, alcoholic liver disease	CF	−	+ Urine	−	*Candida albicans*	HPB infection	Fluconazole	Yes	Yes	3/60
91	49/F/L	DM	6/9	−	−	−	–	Skin infection	Ciprofloxacin, fluconazole	Yes	Yes	HM
92	41/F/R	Leukemia	6/7.5	+	−	Not done	*Candida tropicalis*	–	Voriconazole	Yes	No	6/9
93	41/F/L	Leukemia	6/60	+	−	−	*Candida tropicalis*	–	Voriconazole	Yes	No	6/18
94	58/M/R	DM, liver disease	6/36	−	−	−	–	–	Ceftazidime	Yes	No	6/9
95	55/F/R	Auto-immune hepatitis on steroid and immunosuppressant	CF	−	−	−	–	–	Ciprofloxacin	Yes	No	6/12
96	81/M/R	–	HM	−	−	−	–	–	Ciprofloxacin	Yes	Yes	6/12
97	32/F/R	Leukemia	6/24	−	−	+	*Xylariaceae* sp.	–	Voriconazole	Yes	Yes	CF
98	32/F/L	Leukemia	6/12	−	−	−	–	–	Voriconazole	Yes	Yes	6/12
99	63/F/R	–	HM	−	−	+	*Staphylococcus* coagulase -ve	Skin infection	Ciprofloxacin	Yes	Yes	6/9
100	54/M/L	DM, renal failure	PL	+	+ Infected catheter	+	*Staphylococcus aureus*	Infected catheter	Ciprofloxacin, amoxicillin-clavulanic acid	Yes	No	NPL
101	46/F/R	DM	HM	−	−	+	*Penicillium* sp.	–	Amphotericin, fluconazole	Yes	Yes	CF
102	64/M/L	DM, rectal carcinoma	PL	−	−	−	–	–	Ciprofloxacin	Yes	Yes	HM
103	66/M/L	DM	6/24	+	−	+	*Mycoplasma pneumonia*	Lung infection	Ciprofloxacin, azithromycin	Yes	Yes	6/9
104	59/F/L	DM, renal failure	HM	−	−	−	–	–	Ciprofloxacin	Yes	Yes	CF
105	70/M/R	DM	PL	−	−	+	*Penicillium* sp.	–	Itraconazole	Yes	Yes	6/36
106	41/F/R	DM	HM	−	−	−	–	Lung infection	Ciprofloxacin	Yes	Yes	PL
107	67/M/L	DM	6/9	+	+ Urine	+	Escherichia coli	UTI	Ciprofloxacin	Yes	Yes	CF
108	63/M/R	–	1/60	+	+ Infected catheter	+	MRSA	Skin infection	Vancomycin, ciprofloxacin	Yes	Yes	6/9
109	63/M/L	–	6/24	+	+ Infected catheter	Not done	MRSA	Skin infection	Vancomycin, ciprofloxacin	No	No	6/12
110	52/M/L	DM, renal failure	HM	−	−	+	*Phanerochaete chrysosporium*	Skin infection	Voriconazole	Yes	No	CF
111	45/M/R	DM, renal failure	PL	+	−	−	*Staphylococcus aureus*	Infected catheter	Cloxacillin	Yes	Yes	NPL
112	47/M/R	Alcoholic liver disease on steroid	6/12	+	−	Not done	*Staphylococcus aureus*	Skin infection	Cloxacillin	No	No	6/9
113	47/M/L	Alcoholic liver disease on steroid	6/12	+	−	Not done	*Staphylococcus aureus*	Skin infection	Cloxacillin	No	No	6/9
114	58/M/L	DM	PL	+	+ Infected catheter	+	MRSA	Genital infection	Ciprofloxacin	Yes	Yes	HM
115	67/F/R	–	6/36	−	+ Urine	+	*Klebsiella pneumonia*	UTI	Cefuroxime	Yes	Yes	CF
116	48/M/L	DM	HM	−	−	Not done	–	Skin infection	Ciprofloxacin	Yes	Yes	6/18
117	78/F/L	–	CF	−	−	+	*Staphylococcus* coagulase -ve	–	Ciprofloxacin, ceftazidime	Yes	Yes	3/60
118	61/F/L	DM	PL	−	+ Urine	−	*Klebsiella pneumonia*	UTI	Ciprofloxacin, meropenem	Yes	Yes	CF
119	55/F/L	DM	3/60	+	+ Sputum	−	*Klebsiella pneumonia*	Lung infection	Ceftazidime	Yes	Yes	6/12
120	55/M/L	–	PL	+	−	+	*Klebsiella pneumonia*	HPB infection	Ceftriaxone, ciprofloxacin, metronidazole	Yes	No	NPL
121	60/M/R	DM	PL	+	+ Infected catheter	+	*Staphylococcus aureus*	Skin infection	Cloxacillin, ceftazidime	Yes	No	HM
122	60/M/L	DM	PL	+	+ Infected catheter	+	*Staphylococcus aureus*	Skin infection	Cloxacillin, ceftazidime	Yes	Yes	HM
123	40/F/R	DM/liver cirrhosis, myelodysplastic syndrome	6/18	+	+ Infected catheter	−	*Klebsiella pneumonia*	Lung infection	Cefuroxime, piperacillin-tazobactam	Yes	Yes	CF
124	32/F/L	DM	6/60	+	−	−	*Klebsiella pneumonia*	UTI	Cefuroxime	Yes	No	6/12
125	34/F/L	DM	HM	−	+ Urine	+	*Candida albicans*	–	Amphoterin	Yes	Yes	6/18
126	57/F/R	DM	PL	+	−	−	*Klebsiella pneumonia*	AGE	Ciprofloxacin	Yes	Yes	NAV
127	48/M/R	DM	PL	−	−	−	–	DFU	Ciprofloxacin, Ceftazidime	Yes	No	NPL
128	65/F/R	DM	1/60	−	+ Urine	−	*Klebsiella pneumonia*	UTI	Ciprofloxacin	Yes	No	6/60
129	65/F/R	DM, renal failure	HM	−	−	−	–	–	Ciprofloxacin	Yes	Yes	CF
130	72/F/L	DM	HM	−	+ Urine	−	*Escherichia coli*	UTI	Ciprofloxacin, trimethoprime, sulphamethoxazole	Yes	No	NPL
131	55/F/R	DM	HM	+	−	−	*Klebsiella pneumonia*	HPB infection	Ceftriaxone	Yes	Yes	HM
132	67/M/R	Adenocarcinoma of lung and colon	HM	−	−	−	–	Infected catheter	Fluconazole, voriconazole	Yes	Yes	NPL
133	68/F/L	DM, renal failure	CF	+	−	−	*Elizabethkingia meningocepticum*	Infected catheter	Ceftazidime, cefazolin, vancomycin	Yes	No	CF
134	68/F/R	DM, renal failure	6/24	+	−	−	*Elizabethkingia meningocepticum*	Infected catheter	Ceftazidime, cefazolin, vancomycin	Yes	No	6/12
135	61/F/L	DM, renal failure	HM	+	−	−	*Staphylococcus aureus*	Infected catheter	Cloxacillin	Yes	No	6/36
136	44/M/L	DM	PL	+	+ Liver	+	*Klebsiella pneumonia*	HPB infection	Ceftazidime, imipenem	Yes	No	NPL
137	65/M/L	DM, chronic cystitis	CF	−	+ Urine	−	*Klebsiella pneumonia*	UTI	Ciprofloxacin	Yes	Yes	CF
138	59/F/L	DM, renal Failure	HM	−	−	−	–	Skin infection	Ciprofloxacin	Yes	Yes	6/18
139	67/F/L	Hepatolithiasis	PL	+	+ Liver	−	*Klebsiella pneumonia*	HPB infection	Ciprofloxacin, imipenem	Yes	No	NPL
140	76/M/R	DM	CF	−	+ Urine	−	*Candida albicans*	UTI	Fluconazole	Yes	Yes	6/9
141	76/M/L	DM	CF	−	+ Urine	−	*Candida albicans*	UTI	Fluconazole	Yes	Yes	6/24
142	38/F/R	–	HM	−	−	+	*Pseudomonas aeruginosa*	–	Ceftriaxone, ciprofloxacin, metronidazole	Yes	Yes	HM
143	69/M/L	DM	HM	+	+ Urine	+	*Klebsiella pneumonia*	UTI	Cefepime, amoxicillin-clavulanic acid	Yes	Yes	1/60

*Abbreviation*: *IVT* intravitreal, *abk* antibiotic, *DM* diabetes mellitus, *NAV* not available, *UTI* urinary tract infection, *IE* infective endocarditis, *HPB* hepatobiliary, *DFU* diabetic foot ulcer, *AGE* acute gastroenteritis, *MRSA* methicillin-resistant *Staphylococcus aureus*, *CF* counting finger, *HM* hand movement, *PL* perception of light, *NPL* non-perception of light

**Fig. 4:**
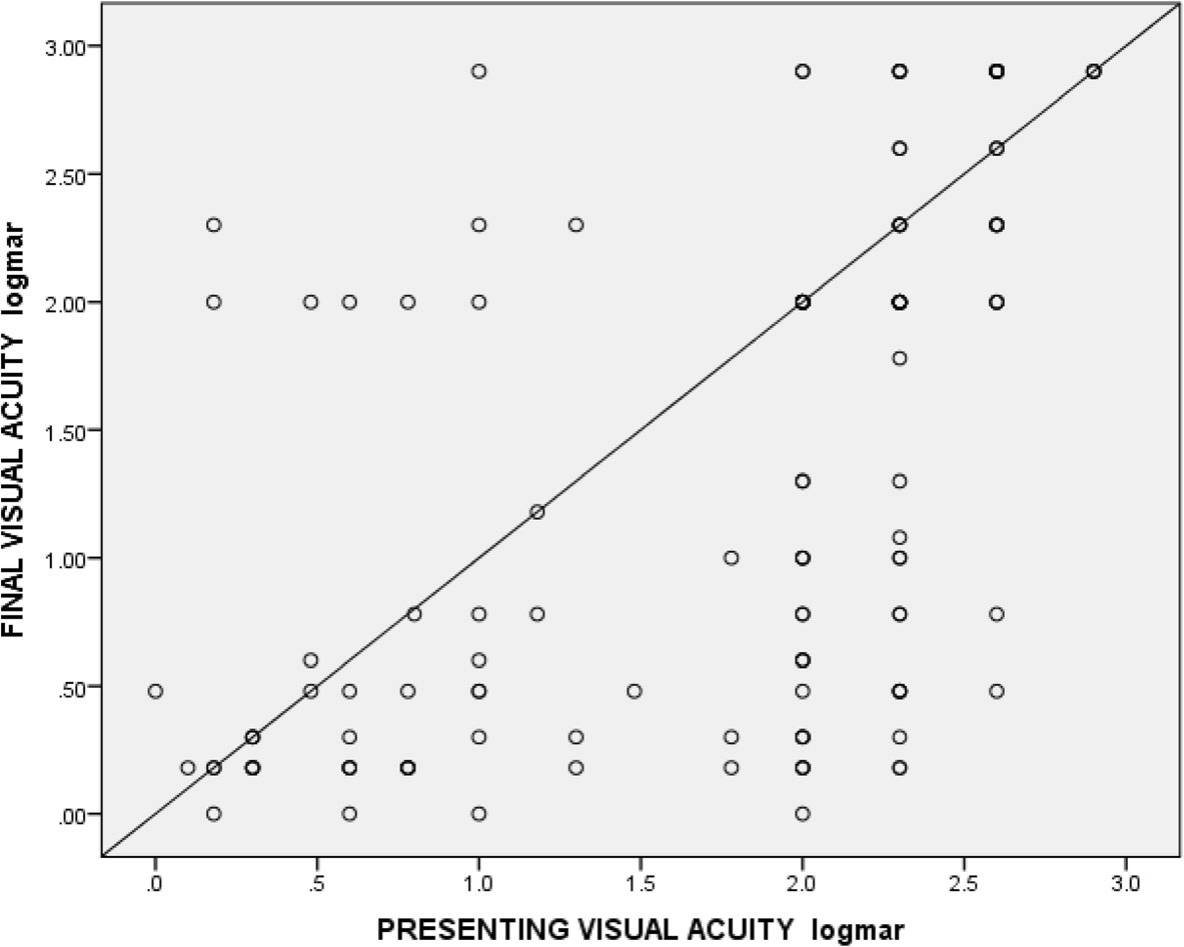
Correlation between presenting visual acuity (LogMAR) and final visual acuity (LogMAR)

In univariate logistic regression analysis, factors found to be statistically significant with good visual outcome were good presenting visual acuity (crude odd ratio 0.1; 95% CI 0.021, 0.384) and presence of fundus view at presentation (crude odd ratio 0.337; 95% CI 0.068,1.675). In the multivariate logistic regression analysis, elevated risk for good visual outcome was observed only in good presenting visual acuity (adjusted odd ratio 0.09; 95% CI 0.021, 0.384). We also found a moderate correlation between presenting visual acuity and final visual acuity (Pearson *r* = 0.564, *p* < 0.001 (Fig. 4).

Gender, age group, presence of underlying medical illness, existing DM, source of infection, culture positivity, types of organism, intravitreal antibiotics, vitrectomy, or early vitrectomy were not significantly associated with good final visual outcomes.

## Discussion

In this study, we wanted to determine whether the clinical profiles of EE at a tertiary hospital in Malaysia were similar to those reported from other countries.

Previous studies had reported a male preponderance with unilateral involvement [7–10]. In contrast, our results showed no difference between male and females.

Predisposing conditions are important in determining a patient’s risk for endogenous endophthalmitis. Okada et al. reported 90% of patients had a positive history of underlying medical conditions such as diabetes, cardiac disease, and malignancy [2]. A major review of endogenous endophthalmitis demonstrated underlying medical conditions predisposing to ocular infection in 56 to 68% of cases [4]. Another study conducted by Wu and colleagues revealed the identification of preexisting predisposing condition in 90.9% of patients, and the most common systemic condition found was diabetes mellitus (50%) [11]. In contrast, Connell et al. reported that intravenous drug abuse was the most common risk factor [1]. Several East Asian studies reported that diabetes mellitus was the most common, and hepatobiliary disease was the second most frequent underlying disease [5, 12–14]. In a review of 57 cases of endogenous endophthalmitis in Korea, diabetes mellitus (46.5%) was the most common underlying disease followed by liver cirrhosis (20.9%) [15]. In a recent study in which all patients had one or more preexisting medical conditions, the most common was also diabetes mellitus (61.9%) [16]. Our series revealed similar results, in which diabetes mellitus was the most common systemic disease (60.0%) followed by renal failure and malignancy. However, liver diseases were identified only in 6 patients.

In a review of cases by Wong et al., it was reported that hepatobiliary tract infection was the most common source of bacteremia (13 patients, 48%) [5]. Similar results were found in other Korean case series [12, 15, 17]. Interestingly, our case series did not show similar findings with other East Asian reports. We found that urinary tract infection (21, 17.5%) was the most common source of bacteremia followed by pulmonary infection (19, 15.8%). Hepatobiliary tract infection was only identified in 12 patients (10.0%). We also found that among patients who were younger than 40 years old, and older than 60 years old, the most common systemic infection was urinary tract infection at 17.4% and 30.0% respectively. In contrast, lung infection (17.5%) followed by hepatobiliary infection (14.0%) was the commonest infections among those aged from 40 to 60 years old.

In a case series by Lim et al., the most common presenting complaint was decreased vision (68.8%) followed by ocular discomfort (44%), red eye (20.8%), and ocular pain (17.4%) [15]. Ratra et al. also reported that reduced vision (60, 98.4%), redness (47, 77%), and pain (42, 68.8%) were the three most common presenting symptoms [8]. Similar to these studies, our study too revealed blurring of vision, eye redness, and eye pain or discomfort as the main presenting ocular symptoms. However in a case series by Nishida et al., floaters was the second most common ocular symptom after blurring of vision [16].

Ratra et al. in their case series demonstrated that all eyes had severe diffuse endophthalmitis involving the posterior pole. Diffuse vitreous exudates were seen in 47 eyes (77%). Retina could be visualized in 13 eyes (21.3%), and 3 (4.9%) had retinal detachment. None had panophthalmitis [8]. In another case series in 18-year review of culture-positive cases in 34 affected eyes, the most common findings were decreased visual acuity (91.1%), vitritis (79.4%), conjunctival injection (67.6%), iritis or retinitis (61.7%), hypopyon (35.2%), and retinal detachment (5.8%) [18]. In our study, 27 eyes (19%) had hypopyon, 64 eyes (44.8%) had fundus view, and 8 eyes (8.2%) were noted to have retinal detachment on ultrasound. Lower percentage of hypopyon in our patients could be due to the application of topical steroids and antibiotics by the referring ophthalmologist.

The diagnosis of endogenous endophthamitis is typically made following microbiologic evidence of infection from intraocular samples (aqueous or vitreous). Positive cultures from the blood, cerebrospinal fluid, or any extraocular site can be highly suggestive. In our series, the organism causing endophthalmitis was identified by a positive culture from at least one body fluid source in 82 patients (68.9%). Blood culture positivity rate varies widely, from 33 to 94% [4, 19]. Previous large case series have shown higher rates of positivity following blood cultures as compared to vitreous aspirates possibly due to a larger volume sampled [2, 4, 11]. In contrast, Ratra et al. had reported that ocular fluid samples tended to give positive culture results more than blood (58.6% vs 3.4%). This is because all the patients with suspected endogenous endophthalmitis immediately underwent an aqueous tap in the outpatient department before any intravitreal therapy [8]. High rate of positive cultures from intraocular specimens was also demonstrated by Okada et al. (86%), Binder et al. (70%), and Ness et al. (81%) [2, 20, 21]. Vitrectomy has a higher diagnostic yield for culture (92%) compared to a vitreous aspirate (44%) [22]. Vitreous samples during vitrectomy were taken near the retinal surface, which can potentially explain the lower yield of needle biopsy. This is because early or localized infection located near the retinal surface might be missed by a needle biopsy [23]. We noted low vitreous yield of organisms in our study. This could be because some of our patients with systemic infection were initially managed by physicians depending on the source of infection. During the time of referral, most of them were already on systemic antibiotics or partially treated. Furthermore, the diagnosis may have been delayed in some while others were generally not stable for early vitreous tapping. Thirty-six patients (30.0%) in our series were culture negative.

Causative organisms vary geographically. Studies from the western population revealed that fungal infection was the main source in predisposed states, such as intravenous drug abusers and immunocompromised patients [1, 19, 21]. In contrast, gram-negative microbes as the causative organisms were overwhelming in the East Asian experience. In these Asian populations, *Klebsiella* was found to be responsible for approximately 90% of all endogenous bacteria endophthalmitis cases [5]. Studies that were conducted in Korea showed that liver abscess was the most common infection source and *Klebsiella* was the most common causative agent [15, 17]. A study from Japan in 2015, however, demonstrated that gram-positive organisms were more common (76.2%) than gram-negative (19.0%), contrasted to the findings from other East Asian studies [16]. *K*. *pneumonia* which is predominant in East Asia may be due to the high incidence of cholangiohepatitis. Therefore, the East Asian population is more prone to have liver abscess than Caucasians [24]. We found that gram-negative organisms were responsible for half of the cases of endogenous endophthalmitis in our case series (42 patients, 50.6%) in which *K*. *pneumonia* was the most common organism isolated (27 patients, 32.5%). Interestingly, in contrast to several East Asian studies, urinary tract infection including renal abscess (9 patients, 33.3%) was the most common source of infection caused by *K*. *pneumonia* followed by lung infection (8 patients, 29.6%) in our series. Liver abscess was identified in 7 patients (25.9%). Necrotizing fasciitis, infected wound breakdown, and acute gastroenteritis (AGE) were noted in one patient each. Apart from that, there was a relatively higher frequency of gram-positive cocci and fungal infection in our study, 32.5% and 16.9% respectively.

Most systemically administered antimicrobials that have been used in the therapy of endophthalmitis do not penetrate well into the non-inflamed vitreous humor. However, the penetration of several antibiotics into the eye may be increased by inflammation which occurs following surgery, trauma, or infection. Kowalski and colleagues compared the minimum inhibitory concentration (MIC) of bacterial isolates from 66 patients with endophthalmitis and found that all of the gram-negative isolates would have been inhibited by levels of ciprofloxacin achievable following systemic administration [25].

In endogenous endophthalmitis, the rationale for use of intravitreal injections as an adjunct to intravenous therapy is also because of reduced permeability of the retinal-pigmented epithelium to systemically administered drugs [26]. Yonekawa et al. showed that early administration, e.g., within 24 h, was associated with a favorable visual outcome [27]. Most of our patients received intravitreal injections within 24 h of diagnosis.

Vitrectomy serves as a diagnostic and therapeutic option. It is indicated in cases with severe vitreous opacities, diffuse retinal infiltration, and poor presenting visual acuity and when there is no clinical improvement with systemic and intravitreal therapy. However, the role and timing of vitrectomy remain unclear in patients with endogenous endophthalmitis. Sheu et al. reported no significant relationship between vitrectomy and visual outcome in *Klebsiella* endophthalmitis. However, they suggested early vitrectomy should be considered in patients whose anterior chamber inflammation did not respond well to intravitreal antibiotics [28]. On the other hand, Yoon et al. demonstrated that following early vitrectomy for *Klebsiella* endogenous endophthalmitis, 50% achieved a vision of CF or better after 6 months [14]. Early vitrectomy performed within 10 days of the appearance of ocular symptoms or signs resulted in a better visual prognosis (CF or better) than without vitrectomy [17]. In other studies, early vitrectomy within 2 weeks of presentation in severe cases or suspected virulent organisms was associated with good overall outcome [14, 17]. In our case series, 73 eyes (51.4%) underwent vitrectomy. Vitrectomy was performed within 2 weeks in 38 eyes (52.1%) and more than 2 weeks in 35 eyes (47.9%). The most common indication for early vitrectomy was poor presenting visual acuity of CF or worse in 31 cases (81.6%). Persistent or increased vitreous opacities or anterior chamber cells despite systemic and intravitreal antibiotics were other indications for early vitrectomy. There was no significant difference between early vitrectomy (within 2 weeks) compared to delayed vitrectomy (more than 2 weeks) for favorable visual prognosis (*p* = 0.327).

Generally, the visual outcome of endogenous endophthalmitis is poor due to early and extensive retinal involvement. Virulent causative organisms, poor host defense, misdiagnosis leading to delayed treatment, inadequate treatment, inappropriate therapy, and occurrence of complications such as panophthalmitis are associated with poor prognosis. Wu et al. reported that the eyes with bacterial endogenous endophthalmitis had a worse outcome compared to patients with fungal endophthalmitis [11]. Lim et al. concluded that gram-negative bacteria had worse visual outcomes compared to gram-positive bacteria or fungus [15].

Visual outcomes in *Klebsiella* endophthalmitis has been poor despite treatment with a combination of systemic and intravitreal antibiotics [12, 13]. Case series and literature reviews involving infection with *K*. *pneumonia* showed that visual acuity achieved was CF or better in 34.0% of eyes, and 16.0% had evisceration or enucleation [5]. Sheu et al. reported 19 eyes (35.8%) had final visual acuity of CF or better [28]. Connell et al. found that all the patients in their study needing enucleation were infected by *Klebsiella* [1]. In our series, 100 eyes (73.0%) achieved final visual acuity of CF or better. However, in cases with *Klebsiella* endogenous endophthalmitis, only 18 eyes (25.4%) achieved final visual acuity of CF or better, which is comparable with other studies. Ten eyes were complicated with panophthalmitis, and 5 of them were due to *Klebsiella pneumonia*.

In our series, a good presenting visual acuity was the only prognostic factor associated with good visual outcomes of CF or better. Lim et al., Nishida et al., and Binder et al. in their case series also described that a good presenting visual acuity was significantly associated with good final visual acuity [15, 16, 20]. We found that DM, presence of a source of infection, organism, and intravitreal antibiotics were not related to poor visual outcome.

### Study limitation

This study is limited by the retrospective design. As the data was collected retrospectively, some of the information was not available. Apart from that, patients with culture-negative result were also included in this study which may have included those with non-infectious uveitis. In the future, we may need to use other methods such as polymerase chain reaction (PCR) with higher sensitivity and specificity. Lack of uniform guidelines and treatment protocol is another limitation. Observational and prospective case series are needed in the future to assess long-term outcomes.

## Conclusions

The visual prognosis of endogenous endophthalmitis (EE) is poor. Gram-negative organisms specifically *Klebsiella pneumonia* were the most common organisms isolated. Urinary tract infection was the main source of infection. Poor presenting visual acuity was significantly associated with poor visual outcomes.

## Abbreviations

AGE: Acute gastroenteritis
CF: Counting fingers
EE: Endogenous Endophthalmitis
MRSA: Methicillin-resistant *Staphylococcus aureus*
PCR: Polymerase chain reaction
